# Challenges and Opportunities Modeling the Dynamic Tumor Matrisome

**DOI:** 10.34133/bmef.0006

**Published:** 2023-01-16

**Authors:** Shelly R. Peyton, Manu O. Platt, Edna Cukierman

**Affiliations:** ^1^ Department of Chemical Engineering, University of Massachusetts Amherst, Amherst, MA, USA.; ^2^ Coulter Department of Biomedical Engineering, Georgia Institute of Technology and Emory University, Atlanta, GA, USA.; ^3^ Cancer Signaling & Microenvironment Program, Marvin and Concetta Greenberg Pancreatic Cancer Institute, Fox Chase Cancer Center, Temple Health, Philadelphia, PA, USA.

## Abstract

We need novel strategies to target the complexity of cancer and, particularly, of metastatic disease. As an example of this complexity, certain tissues are particularly hospitable environments for metastases, whereas others do not contain fertile microenvironments to support cancer cell growth. Continuing evidence that the extracellular matrix (ECM) of tissues is one of a host of factors necessary to support cancer cell growth at both primary and secondary tissue sites is emerging. Research on cancer metastasis has largely been focused on the molecular adaptations of tumor cells in various cytokine and growth factor environments on 2-dimensional tissue culture polystyrene plates. Intravital imaging, conversely, has transformed our ability to watch, in real time, tumor cell invasion, intravasation, extravasation, and growth. Because the interstitial ECM that supports all cells in the tumor microenvironment changes over time scales outside the possible window of typical intravital imaging, bioengineers are continuously developing both simple and sophisticated in vitro controlled environments to study tumor (and other) cell interactions with this matrix. In this perspective, we focus on the cellular unit responsible for upholding the pathologic homeostasis of tumor-bearing organs, cancer-associated fibroblasts (CAFs), and their self-generated ECM. The latter, together with tumoral and other cell secreted factors, constitute the “tumor matrisome”. We share the challenges and opportunities for modeling this dynamic CAF/ECM unit, the tools and techniques available, and how the tumor matrisome is remodeled (e.g., via ECM proteases). We posit that increasing information on tumor matrisome dynamics may lead the field to alternative strategies for personalized medicine outside genomics.

## The Grand Challenges in Studying Tumor Stroma

**Fig. 1.**
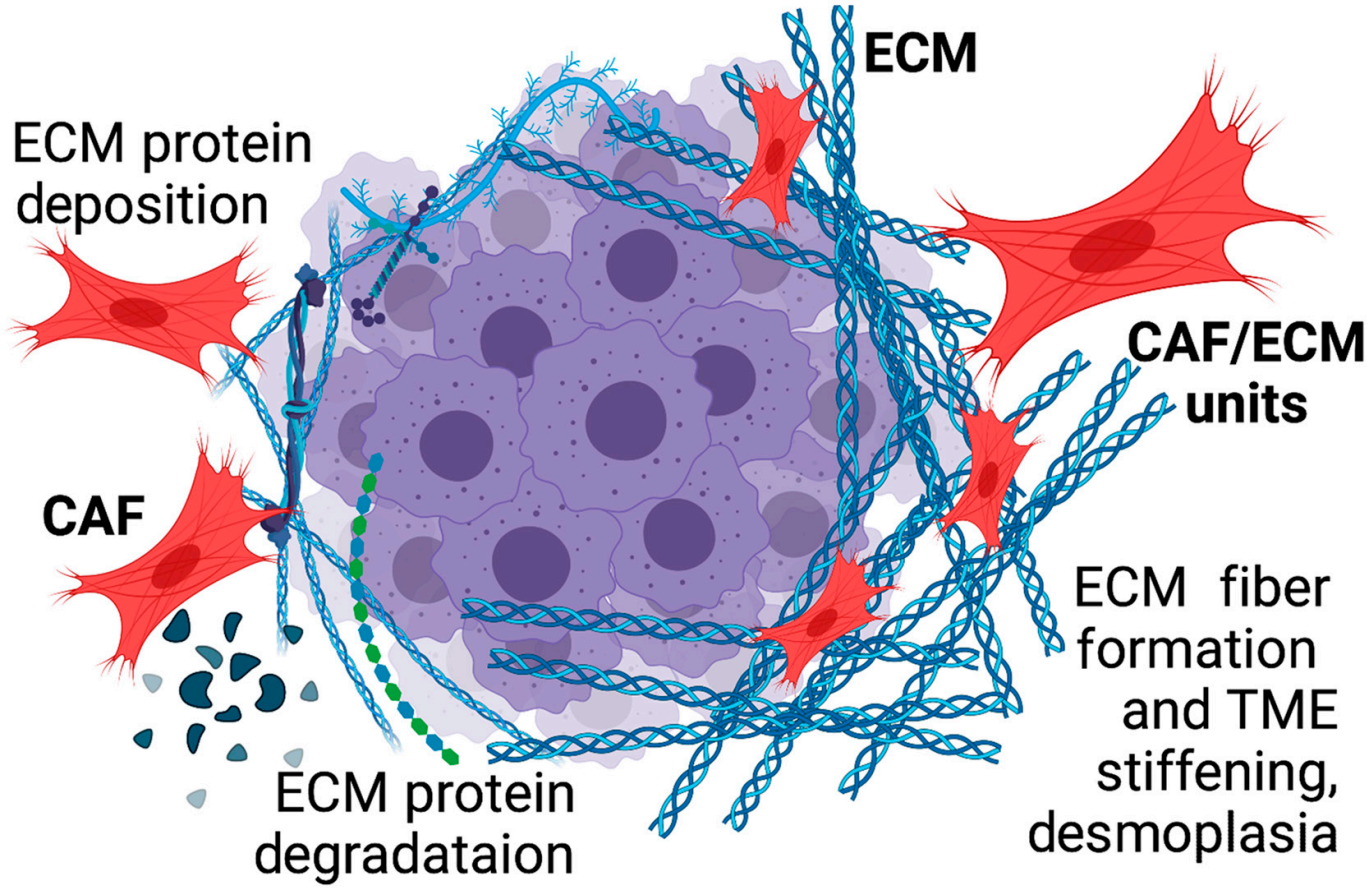
Cancer-associated fibroblasts (CAFs) and their self-generated ECM constitute the desmoplastic functional unit responsible for upholding the cancerous tissue homeostasis. *Created with BioRender.com*

Fibroblastic cells and their self-generated extracellular matrix (ECM) constitute a physiologic functional unit [1]. This basic connective tissue unit sustains the homeostasis of healthy organs and is responsible for orchestrating local and systemic responses to temporary perturbances like wounding or acute infections. Similarly, tumor-educated cancer-associated fibroblasts (CAFs) and the ECM they synthesize constitute a perturbed functional unit that upholds cancer homeostasis (Fig. 1) [2].

Cancer is one of the leading causes of deaths in the US [3]. The majority of nonhereditary carcinomas, like lung, pancreas, liver, colorectal, breast, prostate, and others, develop from epithelial cells that undergo a series of oncogenic mutations. Nonetheless, somatic mutations alone do not fully explain epithelial tumor development. This is probably because this type of tumor onset and its progressive development necessitates that the naturally tumor-suppressive stroma is altered in a way that ceases to restrain tumorigenesis [4–6]. Hence, the noted “full organ” involvement in cancer development suggests that the Somatic Mutation Theory [7,8], arguing in favor of oncogenic-driver mutations, and the Tissue Organization Field Theory [9–11], proposing that the stroma constitutes a natural tumor suppressor in need to be modulated to support tumor development, are mutually inclusive and can collectively explain the local and systemic aspects of tumorigenesis [4]. This idea can begin to explain the reason why numerous attempts to eliminate the stroma have provided no benefit to patients and occasionally have even been detrimental [12].

Importantly, the above-mentioned CAF/ECM functional unit is the main stroma component of solid epithelial cancers, known as desmoplasia [13]. Desmoplasia, described as a chronic healing-like fibroblastic stroma field expansion, favors tumorigenesis by nurturing tumor cells and concurrently sustaining pro-tumor immunosuppression [13]. Nonetheless, while normal stroma is naturally tumor-suppressive, tumor perturbed stroma is both tumor-supportive and tumor-suppressive [13]. Therefore, efforts to inhibit the former while harnessing the natural tumor-suppressive functions of desmoplasia justify ongoing preclinical efforts for attaining an effective “stroma normalization” that could be translated to the clinic.

To this end, since fibroblastic function and its modulation are dictated by the units’ ECM substrate, it is essential to realize that classic, 2-dimensional culturing of fibroblastic cells imposes an artificial type of stress upon these cells. The fibroblastic cell requirement of a pathophysiologic microenvironment for in vivo-like function substantiates efforts for culturing these cells within carefully selected matrix substrates, such as self-generated ECM [1]. Better understanding of the natural intact and cancer-perturbed interstitial ECMs should provide guidance for effective bioengineering of ECM-like substrates with high pathophysiologic relevance [14–16]. Furthermore, studying how intact ECMs are altered to become perturbed ECMs that sustain cancer homeostasis is also of paramount importance [17–19].

Knowing the central role that fibroblastic cells play in wound healing and understanding that sustained healing biases cancer development [20], it is posited that chronic assaults, such as frequent smoking, stress, obesity, “inflamaging,” and others, alter the fibroblastic cell/ECM in a way that this functional unit ceases to actively suppress tumor development. The possibility to detect tumor-predisposing traits and functions [13,21–24] within this functional unit constitutes a highly sought out endeavor. Since the fibroblastic/ECM functional unit can be modulated by cell-intrinsic and -extrinsic factors, better understanding of this unit is necessary to design how stroma normalization could be attained.

Were all fibroblasts and all matrices in tumors the same, this problem would be easier to solve and to translate across different types of patients. However, cancer is not a disease of conformity. Heterogeneity exists across different patients, from one cancer type to another, within the same cancer dispersed at different organs, and even across the cells within a single tumor. This heterogeneity complicates therapeutic strategies, obviously limits the benefits of targeting drugs to a dominant cancer genotype [25], and likely hinders efficacy of immunotherapy [26]. When including the heterogeneity of the stroma [27], matrix [28], etc., a more complicated environment is considered and opens further experimental pathways to be investigated. Engineers have stepped in to develop systems to modulate the tumor ECM with biomaterials controlling architecture and mechanics as well as varying presentation of synthetic polymers, natural polymers, glycans, and soluble factors [29], which we will discuss here, particularly within the context of the dynamic, continually remodeled CAF/ECM unit.

## Remodeling of the Tumor Stroma

The stroma of solid epithelial cancers, including its scaffolding interstitial ECM, generated and sustained by a reciprocal crosstalk with local CAFs (e.g., CAF/ECM units), constitutes a key feature of tumorigenesis. This is because the tumor-associated stroma can generate barriers and paths, which cage or gate the entrance as well as the escape of key cellular components (e.g., cancer cell metastatic escape or immune cell access). Being both physical and biochemical, these barriers and paths modulate the location as well as the functional states of a plethora of cancer and tumor microenvironment (TME) cells. An important feature of the above-mentioned pathologic homeostasis is explained through the clear increase in tumor and stroma secreted TME modulators. For example, high levels of cysteine cathepsins, matrix metalloproteases, and other proteases [30–36] specialize on cleaving unique components of the epithelial and the interstitial ECMs such as laminin, collagens type I and IV, and fibronectin [37–39]. Proteases can cleave this ECM to make large spaces and overcome physical barriers, thus facilitating the metastatic escape of cancer cells in bulk or as single cells (e.g., through an epithelial-to-mesenchymal transition). Importantly, if the generated gaps do not render sufficient access, cancer cells can still escape by altering their migratory strategy, using a mesenchymal-to-ameboid switch, and squeeze through the protease-generated and natural ECM pores [40].

Of note, dynamic interstitial ECM changes are a noteworthy requirement of in vivo tumorigenesis. To this end, the late Dr. Keely revolutionized the biophysics interstitial ECM field in a series of seminal discoveries. The first demonstrated that the biophysical state of fibrous, collagen-I-rich ECMs (e.g., connective tissue interstitial ECM) dictates epithelial cell function [41,42]. The second revealed that during tumorigenesis, fibroblast/ECM units undergo a dynamic reorganization of collagen I ECM fibrils, which result in the formation of 3 possible types of distinct and measurable “tumor-associated collagen signatures”, known as TACS-1, TACS-2, and TACS-3 [43]. Importantly, Keely (and others after her) demonstrated that patients with surgically resected tumors that encompass high levels of TACS-3 (e.g., ECM fibers that are parallely aligned and oriented perpendicularly to the tumor border) within the TME experience shorter overall survival [44–47]. This increase in the amount and alignment of collagen fibers also stiffens the ECM at the tumor border, from hundreds of pascals to tens or even hundreds of kilopascals [43,48–60].

**Fig. 2.**
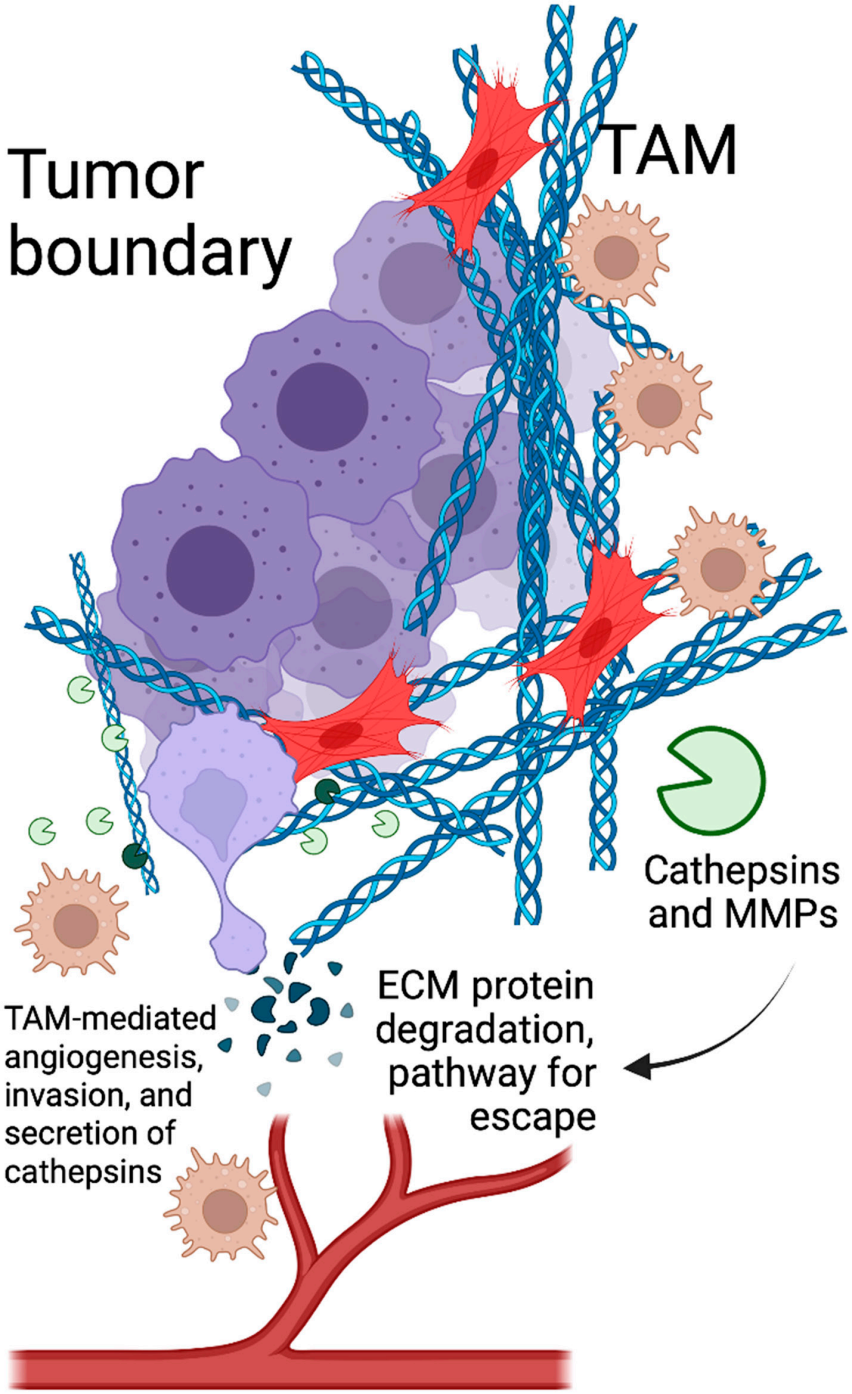
Cathepsins and other proteases released by CAFs and TAMs mediate degradation of ECM at the tumor boundary, leading to cancer cell escape to nearby blood vessels and eventual metastases. Created with BioRender.com

Furthermore, individual matrix proteins have been identified that either suppress overt metastasis, including thrombospondin 1 on the microvascular endothelium [61], or facilitate it, such as in the case of CAF/ECM units generating differentially spliced variants of fibronectin [62–64]. Moreover, tumor cells can trigger stromal periostin secretion to metastasize to the lung [65], and breast cancer cell-derived tenascin C promotes lung metastasis [66]. As other examples, ECM proteins like fibronectin, collagen, and others, as well as their protease-generated fragments, can support the survival of incoming disseminated tumor cells [67,68]. Notably, some of the proteins, including many key chemokines and cytokines that signal to local cancer and stroma cells, are activated or facilitated by protease activity; many are the result of ECM fragmentation, while others are released and often even activated due to ECM cleavage [69] (Fig. 2).

Beyond the tumor cells and the CAF/ECM units, another central player promoting cancer cell escape from the primary tumor site is the tumor-associated macrophage (TAM) [70]. Infiltration of TAMs is associated with poor prognosis [71–75], and at the tumor, TAMs promote angiogenesis [74,76–80], chemotherapy resistance [81], tumor growth [76], invasion, metastasis [82], and tumor-promoting immune responses [83–86], and secrete tissue-remodeling cysteine cathepsin proteases [87–90] and other proteases [91–93] (Fig. 2). TAM-secreted proteases play a critical role at the primary tumor [81,87]. This could implicate targeting of macrophages, or even monocytes, in the marrow milieu as a powerful mechanism to decrease metastatic burden. Studies have also shown that monocyte-derived macrophages as models for TAMs can assist cancer cell invasion in patient-specific ways [94,95].

## Experimentally Modeling the Dynamic TME

Bioengineers are continuously iterating on and refining the experimental systems in which to study the impact of the CAF/ECM unit on tumor growth, progression, metastasis, and drug response, as well as how the tumor matrisome co-evolves with the tumor cells, via remodeling or otherwise. Thus far, the gold standard has been rodent models. The field acknowledges that rodent models do not perfectly reflect human disease [96], yet it is currently not possible to validate findings from in vitro experiments any other way. Rodent models often lack a human-like stromal architecture, have a rate of disease progression that far outpaces that in humans, and do not have a human immune system (some models have a near lack of any immune system) [97].

To bridge the gap between oversimplified plastic dishes and rodent models, bioengineers have been creating increasingly more complex and realistic environments to mimic the human TME. The NCI, originally partnering with the NSF, has established venues to specifically fund this work through the Physical and Engineering Sciences in Oncology program (NSF 12-514). Phenomenal advances have been made in tissue engineering to create both organs and systems of tissues [98,99]. By eventually combining these with tumor studies, the dream is that it may be possible to replace rodent models with these engineered systems and do all cancer work in an entirely human-like dynamic TME system.

Toward these more human-like studies, there is increasing effort to use patient-derived tumoroids, organoids, and ex vivo tumor slices in in vitro cancer research, as they better reflect the genetic makeup of tumors in patients. This field is new, but far more is known about the genetic makeup and selection of cells during organoid culture compared to the makeup of the stroma in these patient-derived organoids. Pioneering work from the Hynes lab has defined the matrisome, including the insoluble scaffolding ECM and other secreted factors, via semiquantitative mass spectrometry and provided substantial detail on the matrisomal stroma of mouse tumors [100], which has been corroborated with gene expression of stroma markers in humans [101,102]. To date, we do not know the composition of the matrisome of patient-derived organoids in in vitro systems, and this will be key to determining if these tumor models are reflective of the real TME. Patient-derived organoids are also an opportunity to better understand sex- and ancestry-driven differences in TME dynamics, which has thus far not been addressed. Given the emerging information on sex- and ancestry-driven differences in cell sourcing and disease [103–105], this is a critical gap in matrisome knowledge. Yet, organoids do not often sustain/include stromal cells and thus their TME remains limited.

Perturbing the matrisome of tumors in vivo is challenging. Many ECM protein deletions are embryonically lethal, and conditional mouse models are not economically or technologically accessible to most laboratories (in the US, let alone globally). The ability to perturb the matrisome on the bench is incredibly valuable for hypothesis testing before moving to preclinical models. Several model systems, in vitro, exist where one could replicate many components of the matrisome and include cancer cells or organoids. Matrigel, despite its drawbacks, remains the most widely used of these in vitro cancer cell culture environments [106]. It is very easy to use and contains nearly all the complexity of the basement membrane, which constitutes an important aspect of the TME matrisome of many solid cancers. The overwhelming frustration with Matrigel is its variability, a weariness that is exaggerated given the high cost. Furthermore, Matrigel in the normal basement membrane does not facilitate invasion, but instead is a barrier for solid cancer invasion. Matrigel contains the proteins from the basement membrane (and many more), but it is typically reconstituted in vitro in a manner not tightly crosslinked like the native basement membrane. The native epithelial basement membrane sustains cancer cell growth and simultaneously provides a barrier for cancer cell invasion. In contrast, the interstitial matrix sustained in vivo by CAF/ECM units that is needed to facilitate metastatic escape is often modeled using fibrillar collagen type 1 gels.

However, other systems, despite development effort across many groups, still fall short of Matrigel and other cell-derived ECMs’ performances. Then, again, polymeric hydrogels are advantageous because they are immensely controllable—the end user can carefully dial-in a desired modulus, integrin-binding proteins or peptides included [14,16], tailor the degradation of the cell-encapsulating material, and control the porosity using leaching techniques or allow cells to move by including protease-cleavable crosslinks [107]. Drawbacks to these hydrogels are that they do not have the 3-dimensional structure of native basement membranes or the 3-dimensional fibrillar topography of interstitial ECMs (e.g., generated naturally by tumor-educated CAFs), the posttranslational modifications on native proteins, or the richness of growth-factor-binding sites typical of sugars and proteoglycans offered by native matrices [108]. Peptide-based hydrogels are controllable and give a fibrillar structure akin to the interstitial ECM (reviewed in [109]). Cell attachment points rely on serum and cell-secreted proteins binding electrostatically to the fibrillar matrices or by incorporating integrin-binding peptide fragments into the sequence. Their use as cancer models has been limited, but if one could build more functionality into the peptides [14], or control secreted proteins to represent that of the CAF/ECM unit, these could become powerful models. Cell-derived ECMs are one of the best alternatives, as they have the natural protein structure, yet they are only tunable if one can control the fibroblastic ECM-secreting and -organizing proteins in culture, as well as the underlying stiffness of the substrate used to attain cell-derived ECM fibrillogenesis [24,110,111]. For example, fibroblasts can be isolated from healthy tissue, tumor-adjacent tissue, or intratumoral CAFs (e.g., educated by the tumor; Fig. 1). Fibroblasts can also be biomechanically, genetically, and/or biochemically modulated (e.g., using defined mixtures of growth factors and cytokines), and thus engineered to produce and organize specific types of ECM presenting altered functions [22,45,110–112].

The immune component of the TME, which is not a focus of this particular perspective, has been studied in vivo, but in vitro experiments are far behind. Macrophages, NK cells, neutrophils, and cytotoxic T cells, all important for tumor cell killing in immunotherapies, are short lived in vivo, and strategies to keep them alive for study in vitro are in development. Ideally, one could study the evolution of immune environment alongside the stroma in vitro to hypothesis test and screen immunotherapies. A major roadblock remains, however, in that it is technically challenging to culture most immune cells (macrophages are an exception) in in vitro cell culture environments. Recent advancements have included those from patient-derived organoids, which include all components of the tumor all at once rather than combining isolated components from different sources (for a more complete review on these systems, see [113]).

Computational and mathematical models can be helpful to integrate these complexities: agent-based models, mathematical models of mechanics incorporating biophysical parameters, analytical models of stochastic dynamics of mutations, and kinetic equations incorporating cell growth and death are examples used to assess and derive predictions of both collective and individual tumor cell responses. Systems biology approaches are used to analyze, integrate, and bridge data derived from in vitro and in vivo approaches [114–118] and simulations of CAF/ECM evolution alongside tumorigenesis [111,119,120]. Feedback loops elicited by small-molecule therapies can, at times, be developed or predicted through merging experimental and computational approaches [17,19,25]. Furthermore, computational models have been able to explain that to account for cell–ECM reciprocal interactions, the interstitial ECM necessitates specific viscoelastic properties that could have not been explained with the previous thinking of these scaffolds as plastic microenvironments [119]. Limitations of computational models in the data are collected to build the model and the correctness of assumptions used in their derivation, but their power lies in the manipulations and simulations that can be simulated to conserve time, reagents, and money incorporating physical, cellular, and environmental parameters.

## Outlook

The ECM of the TME can confer unique survival advantages to cancer cells in the presence of otherwise powerful chemotherapies and targeted drugs. There is particular interest in targeting tumor desmoplasia as a way to kill cancer cells, in addition to, or as an alternative to personalized therapies targeting genetic mutations. First described for hypoxia [121], synthetic lethalities designed to hit the tumorigenic ECM are of increasing interest (reviewed in [122]). An overwhelming need is to study the intersection of CAF/ECM evolution and immunotherapy to increase T-cell infiltration into tumors and potentially understand how the ECM regulates T-cell activation and exhaustion.

It is not yet clear if the concept of genetic (including epigenetic) heterogeneity across tumors, organs, and individuals extends to the ECM. Matrisome studies, in fact, suggest that the desmoplastic ECM presents with commonalities across patients and tumor types. We hence wonder if potentially identifying unifying themes among types of tumors despite their organ location could render means to regulate and/or prevent tumorigenesis. Then, again, the fact that certain chronic conditions can solely predispose patients to develop specific cancers, like in the case of pancreatic cancer linked to chronic pancreatitis, suggests that key differences in stroma could encourage unique tumor onsets. Could this type of particular and common tumor stroma-predisposing information help oncologists reach a broader patient population? After all, themes around the CAF/ECM unit that are ubiquitous (or indicative of predispositions) could assist in reducing patient pools based solely on ultra-targeted, and often very expensive, genetic-motivated therapies. Focusing on the TME (including CAF/ECM functional units) could get us to identify “generic” or at least more inclusive and powerful cross-cutting tools.
